# Conversion hepatectomy for advanced hepatocellular carcinoma after right portal vein transection and lenvatinib therapy

**DOI:** 10.1186/s40792-020-01078-3

**Published:** 2020-12-10

**Authors:** Yuki Ohya, Shintaro Hayashida, Akira Tsuji, Kunitaka Kuramoto, Hidekatsu Shibata, Hiroko Setoyama, Hironori Hayashi, Kazumi Kuriwaki, Masato Sasaki, Masayoshi Iizaka, Osamu Nakahara, Yukihiro Inomata

**Affiliations:** 1 Department of Surgery, Kumamoto Rosai Hospital, 1670 Takehara-machi, Yatsushiro, Kumamoto 866-8533 Japan; 2 Department of Gastroenterology and Hepatology, Kumamoto Rosai Hospital, 1670 Takehara-machi, Yatsushiro, Kumamoto 866-8533 Japan; 3 Department of Diagnostic Pathology, Kumamoto Rosai Hospital, 1670 Takehara-machi, Yatsushiro, Kumamoto 866-8533 Japan

**Keywords:** Hepatocellular carcinoma, Lenvatinib, Hepatectomy, Portal vein transection

## Abstract

**Background:**

Lenvatinib is a novel tyrosine kinase inhibitor that exhibits an antitumor effect on hepatocellular carcinoma (HCC). An established strategy that involves surgery and usage of lenvatinib for advanced HCC remains elusive.

**Case presentation:**

A 58-year-old male patient with advanced HCC and untreated hepatitis B was referred to our hospital. The tumor at the right lobe was 10 cm in diameter with right portal vein thrombus. Because of the possible lung metastasis and concern about the remaining hepatic function after extended right hepatectomy, lenvatinib was initiated before surgery. After the confirmation of a sharp decrease of tumor markers during the 3-week lenvatinib therapy, only a right portal vein transection was done leaving the enlargement of the left lobe for improved post-hepatectomy liver function while lenvatinib therapy was continued. The laparotomy revealed that the tumor was invading the right diaphragm. After 7 weeks of lenvatinib administration after right portal vein transection, an extended right hepatectomy with resection of the tumor-invaded diaphragm was successfully done. The lung nodules that were suspected as metastases had disappeared. The patient has been doing well without any sign of recurrence for 1 year.

**Conclusion:**

The strategy involving the induction of lenvatinib to conversion hepatectomy including the portal vein transection was effective for advanced HCC.

## Background

Hepatocellular carcinoma (HCC) is the third leading cause of death worldwide, with incidence continuing to increase [1, 2]. Due to the heterogeneity of HCC and the recent introduction of new agents like tyrosine kinase inhibitors, the current treatment options remain controversial. Currently, surgical treatment of HCC is the most important means for long-term survival of HCC patients [3]. It is difficult to make a surgical choice for advanced HCC with portal vein invasion, diaphragmatic invasion and lung metastasis [4–6]. According to the AJCC 8th staging system, HCC with diaphragmatic invasion and invasion to the major branch of the portal vein are classified as advanced stage disease [7], and they are associated with a poor prognosis [4–6].

Lenvatinib (Eisai Europe Ltd. the United Kingdom) is an orally administered, multitargeted tyrosine kinase inhibitor that selectively inhibits vascular endothelial growth factor receptor (VEGFR) 1–3, fibroblast growth factor receptor (FGFR) 1–4, platelet-derived growth factor receptor alpha (PDGFRα), rearranged during transfection (RET), and KIT [8–12]. Lenvatinib has been shown to be non-inferior to sorafenib in overall survival in untreated advanced hepatocellular carcinoma [13]. A strategy that combines surgery with lenvatinib for advanced HCC has not yet been established. In this case, we report a case of hepatocellular carcinoma undergoing extended right hepatectomy after right portal vein transection and lenvatinib therapy.

## Case presentation

**Fig. 1:**
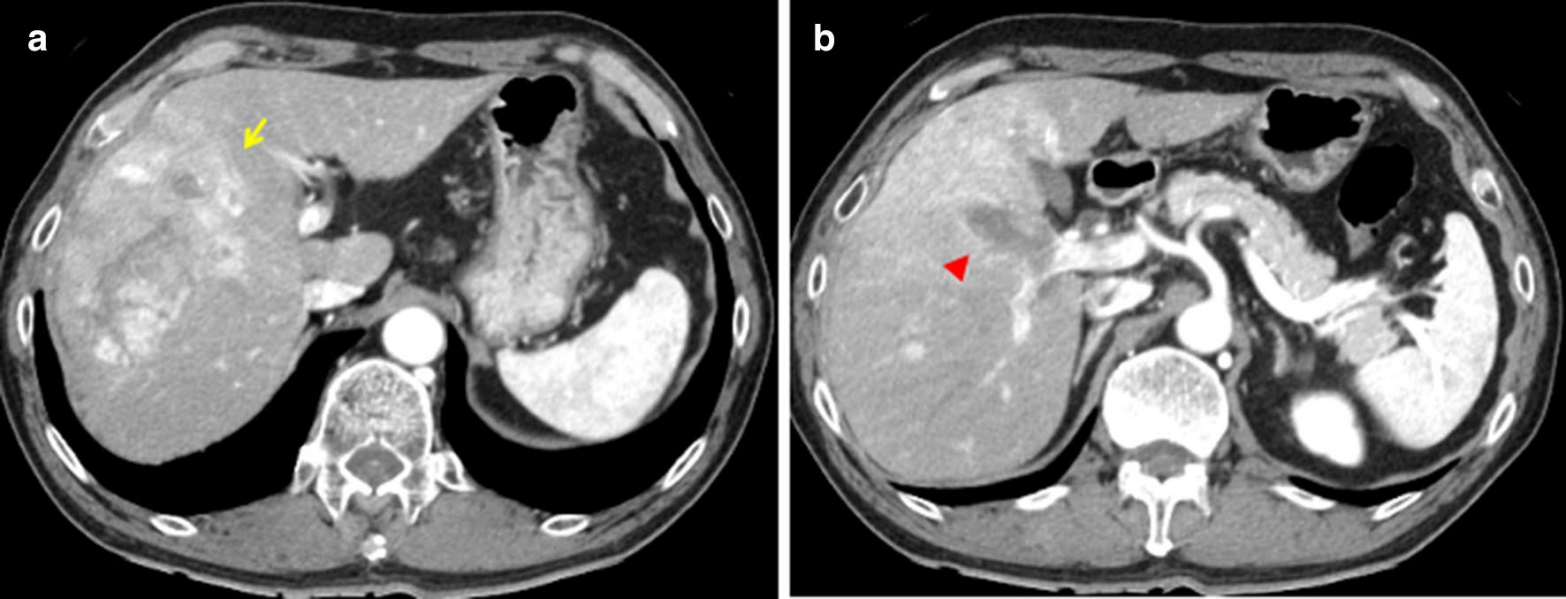
Abdominal enhanced CT findings at the presentation of the patient. **a** CT showing 10 × 7 × 7 cm-sized early enhancement and later washout tumor in the right lobe (yellow arrow). **b** CT showing portal vein invasion or tumor thrombus in the right portal vein (red arrowhead). *CT* computed tomography

**Fig. 2:**
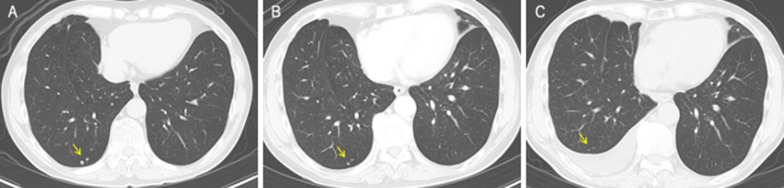
Chest CT findings. **a** CT at the presentation of the patient showing pulmonary nodules in the right lower lobe of the lung suspected to be lung metastasis (arrow). **b** The size of nodules decreased after 2-week administration of lenvatinib (arrow). **c** The size decreased further 3 months after the hepatectomy

A 58-year-old male consulted with his primary doctor regarding right abdominal pain. The patient was referred to our hospital with suspicion of HCC after conventional computed tomography (CT). Contrast-enhanced abdominal CT revealed an approximately 10 × 7 × 7 cm-sized tumor showing early enhancement and later washout with portal vein invasion in the right hepatic lobe (Fig. 1). Chest CT showed pulmonary nodules in the right lower lobe of the lung, and lung metastasis were considered (Fig. 2). Gadoxetic acid-enhanced magnetic resonance imaging (MRI) showed a tumor with heterogenous enhancement on early phase and washout during portal vein phase (data not shown). Laboratory examination showed slight hepatic dysfunction; aspartate transaminase was 57 IU/L (normal 13–30), alanine transaminase was 57 IU/mL (normal 10–42), alkaline phosphatase was 356 IU/mL (normal 106–322), serum albumin level was 4.3 g/dL (normal 4.1–5.1), total bilirubin was 0.75 mg/dL, prothrombin time-international normalized ratio (PT-INR) was 1.02 (normal 0.85–1.15), and the ICG retention rate at 15 min was 10.2% (normal ≤ 10%). Serology for hepatitis virus showed that the hepatitis B surface antigen was positive, anti-hepatitis C antibody was negative, and the hepatitis B virus (HBV) DNA was found to be 1.3 Log IU/mL. The levels of tumor markers before initial treatment were high; alpha-fetoprotein (AFP) level was 74.4 ng/mL (normal ≤ 13.4) and protein induced by the absence of vitamin K or antagonist-II (PIVKA-II) was 1204.58 mAU/mL (normal ≤ 40).

**Fig. 3:**
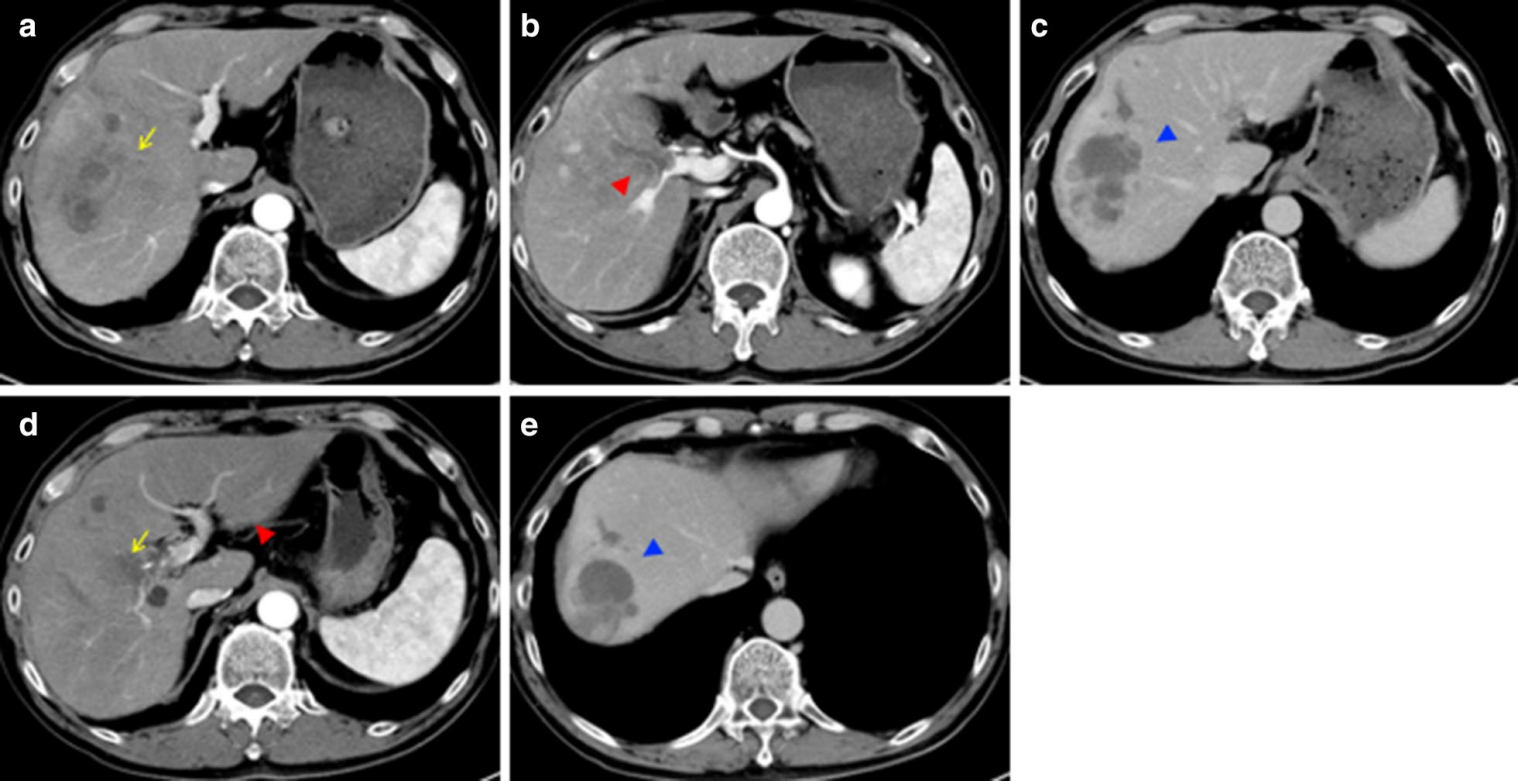
Abdominal enhanced CT findings in the course. **a**–**c** After two weeks of lenvatinib administration before the portal vein transection. CT showed reduction of tumor vascularity (yellow arrow), and extension of portal vein invasion (red arrowhead), as well as tumor necrosis (red arrowhead). **d**, **e** One month after the right portal vein transection. Absent flow in the right portal vein (yellow arrow), enlargement of the left lateral segment (red arrowhead), and progression of tumor necrosis (blue arrowhead)

**Fig. 4:**
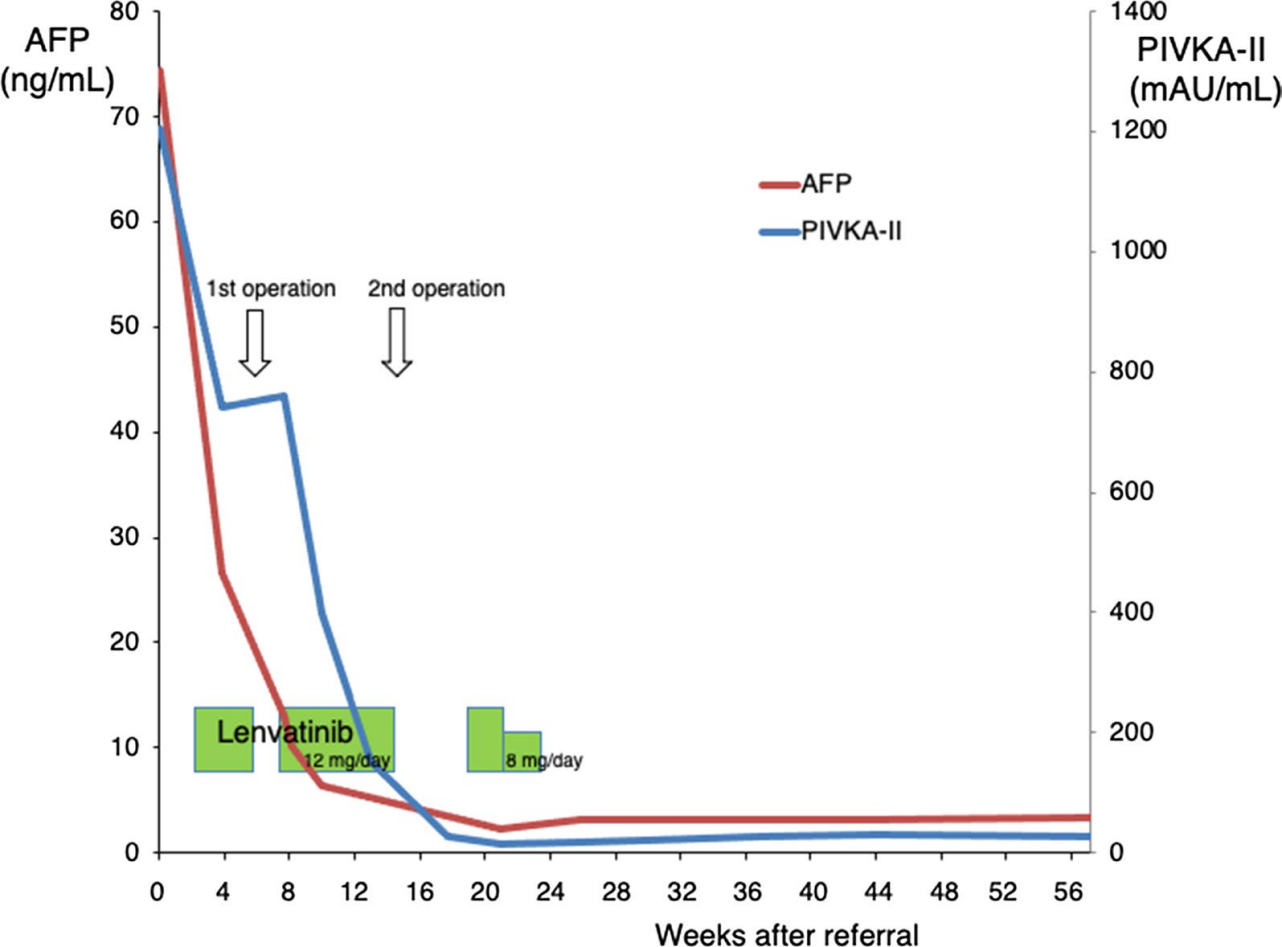
Perioperative changes in AFP and PIVKA-II levels. Levels of AFP and PIVKA-II sharply decreased with lenvatinib administration and returned to normal values after the hepatectomy. AFP and PIVKA-II remained within the normal range after discontinuation of lenvatinib

**Fig. 5:**
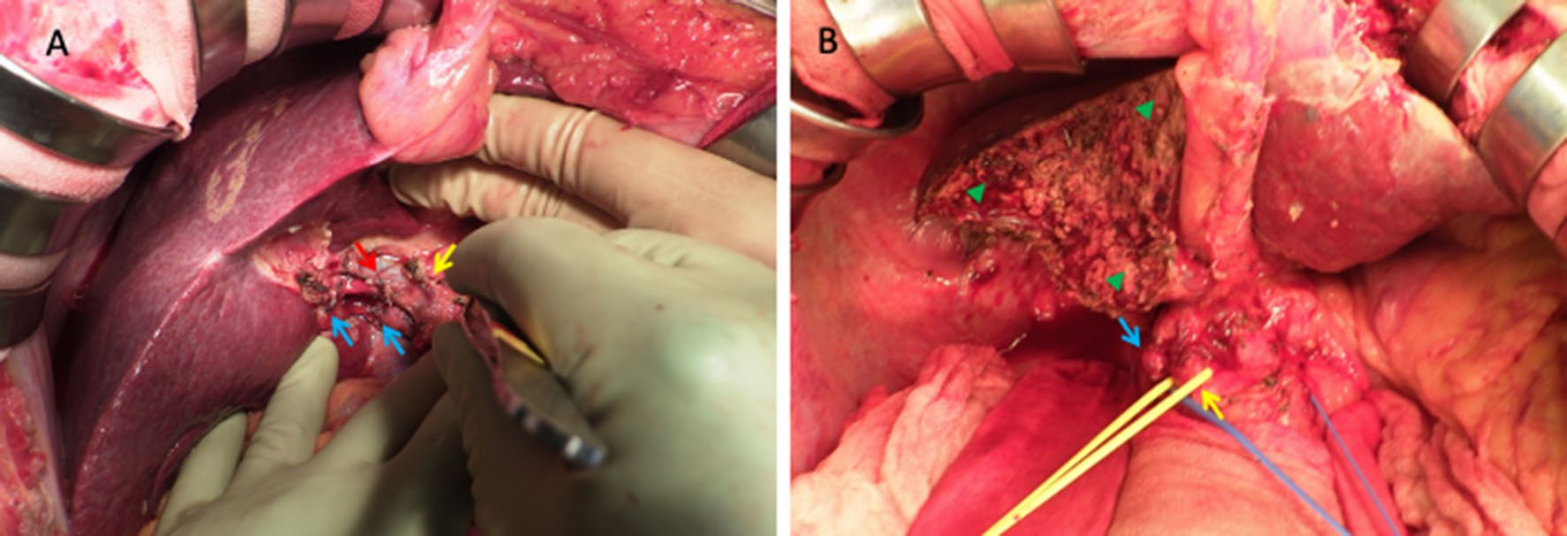
Surgical images. **a** The operation of right portal vein transection. Light blue arrows show the stumps of right portal vein. Red arrow shows the stump of cystic artery. Yellow arrow shows the stump of cystic duct. **b** The operation of conversion hepatectomy. Light blue arrow shows portal vein. Yellow arrow shows common bile duct. Green arrow heads show cutting surface of partial resection of the left medial segment

**Fig. 6:**
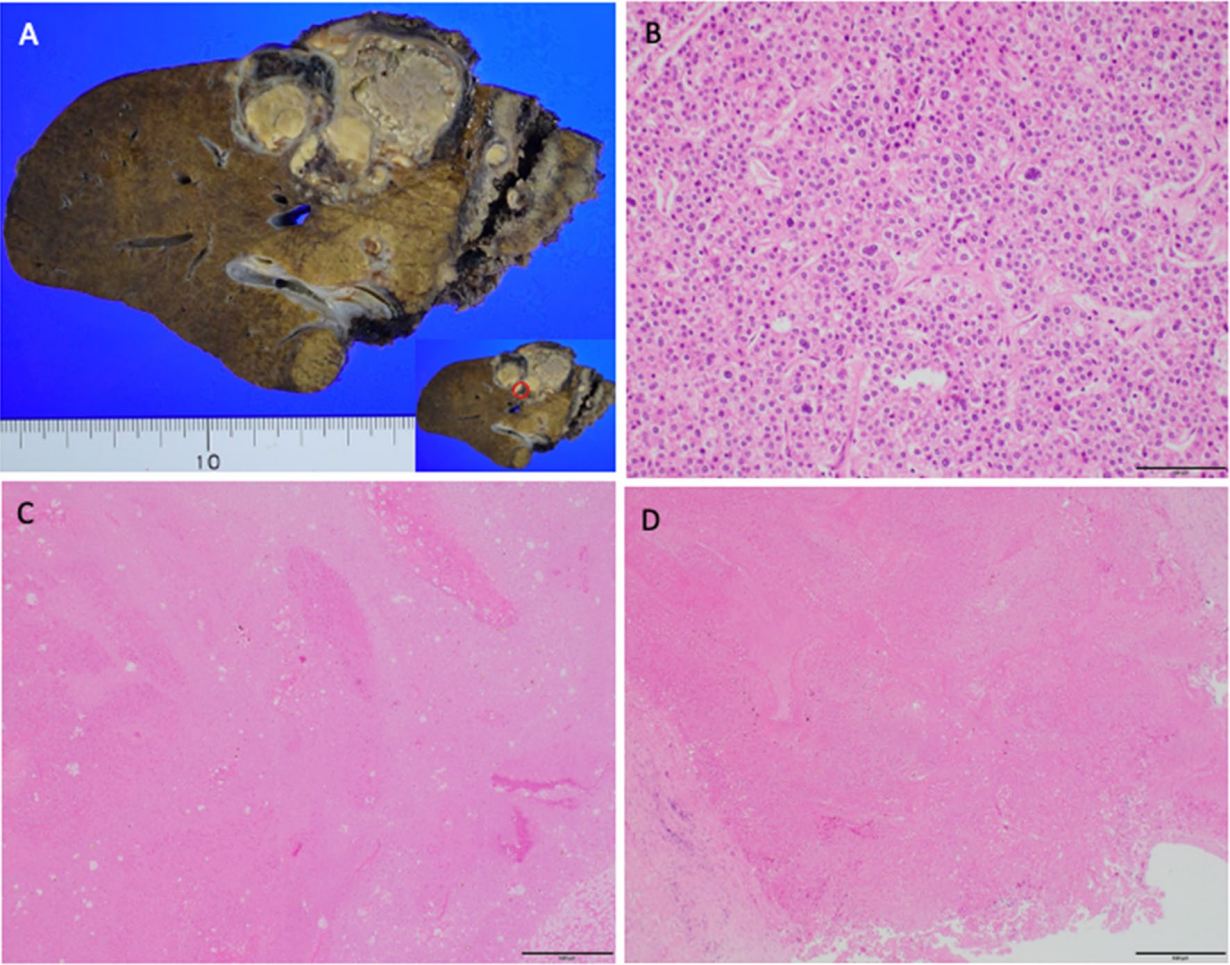
Pathological findings. **a** Cut surface of the formalin-fixed liver with solid masses in the right lobe. The part surrounded red circle included viable carcinoma. **b** Hematoxylin and eosin (HE) staining showing viable cancer cells in the liver tumor. Magnification, ×100. Scale bar: 200 μm. **c** HE staining showing necrosis of the tumor. Magnification, ×40. Scale bar: 500 μm. **d** HE staining showing tumor necrosis of portal vein invasion without viable cancer cells. Magnification, ×40. Scale bar: 500 μm

Considering the lung metastasis and the large right lobe tumor invading the right portal vein, chemotherapy was selected over primary surgery. Lenvatinib (12 mg/day) was applied for two weeks with an anti-HB agent (tenofovir alafenamide). Re-evaluation with contrast-enhanced CT after 2 weeks of lenvatinib administration showed tumor necrosis, the reduction of tumor vascularity, slight shrinkage of pulmonary nodules, and a little extension of portal vein invasion (Figs. 2, 3). Grade 2 hypertension was observed 7 days after the initiation of lenvatinib therapy. Levels of AFP and PIVKA-II sharply decreased (Fig. 4). From the findings of the portal vein invasion, we were concerned about the possible extension of the invasion to the contralateral branch and then loss of the chance of hepatectomy. Therefore, we decided to do portal vein transection and continue further lenvatinib therapy after that. Even at this moment, the estimated liver remnant volume was not enough (estimated liver remnant volume: 482 mL, 40.3% of total liver volume), and we were reluctant to do an extended right hepatectomy. He underwent a right portal vein transection after 1-week withdrawal from lenvatinib intending for the enlargement of the left lobe while more lenvatinib therapy were added (Fig. 5). The laparotomy revealed invasion to the right diaphragm. Enhanced abdominal CT showed left lobe hypertrophy and progression of tumor necrosis 1 month after the transection of the right portal vein (Fig. 3). Estimated liver remnant volume after extended right hepatectomy was 42.1% of total liver volume. Although estimated liver volume showed only a slight increase, we decided to perform the operation not to lose the chance of conversion hepatectomy. He underwent an extended right hepatectomy with partial resection of the left medial segment and a part of the right diaphragm 2 months after the initial right portal vein transection (Fig. 5). The resected tumor was 5.2 × 3.6 cm in size, and the pathological diagnosis was moderately differentiated hepatocellular carcinoma (pT4pN0M0 pStage IVA, vp3, vv0, b0, p0 UICC 8th) (Fig. 6). Intraoperative irrigation cytology was negative, and the resected diaphragm and tumor of right portal vein showed necrotic changes of the carcinoma as well as the absence of viable carcinoma. Non-cancerous liver tissues were noted to be A1F2 based on New Inuyama Classification [9]. The level of AFP and PIVKA-II returned to normal values after the hepatectomy. Although lenvatinib was resumed 1 month after the second operation, he developed grade 2 thrombocytopenia 2 weeks after resumption. This adverse event prompted the halting of lenvatinib administration 1 week after its reduction (Fig. 4). Platelet count recovered immediately after halting of lenvatinib. Even after discontinuation of lenvatinib, AFP and PIVKA-II remained within the normal range (Fig. 4). Chest CT showed a decrease in size of the nodules after 3 months (Fig. 2) and subsequent disappearance 6 months (data not shown) after hepatectomy. At 9 months after the hepatectomy and 1 year after diagnosis, the patient is doing well with no tumor recurrence.

## Discussion

In this case, the patient with HCC showed portal vein invasion (vp3), diaphragmatic invasion, and suspected lung metastasis. Hypertrophy of the future liver remnant by right portal vein transection while continuing lenvatinib therapy was considered to be a strong possibility. Finally, the patient was considered a viable candidate to undergo curative conversion hepatectomy. Lung metastasis had been the target of additional resection after hepatectomy, but they fortunately disappeared in the postoperative CT. There are some reports about conversion hepatectomy for advanced hepatocellular carcinoma after lenvatinib therapy [10–16]. Sato et al. and Tomonari et al. reported conversion hepatectomy for HCC after transarterial chemoembolization (TACE) and lenvatinib therapy [10, 11]. Matsuki et al. reported a complete pathological response in conversion hepatectomy from lenvatinib treatment for advanced hepatocellular carcinoma [12]. Chen et al. reported conversion hepatectomy after lenvatinib therapy in combination with nivolumab [13]. The patients in these reports and our patient underwent conversion hepatectomy while the lenvatinib had effect on HCC. This suggests that surgery should be tried while lenvatinib remains effective for control of the tumor growth. Tumor marker like AFP or PIVKA-II would be indicators for the effectiveness of the drug. In fact, it is difficult to expect the timing of the re-increase of the markers. When the findings of the tumor are suppressed in the imaging studies for safer and complete resection while the tumor markers remain suppressed, it will be the good timing to concert to hepatectomy. Therefore, we need to follow-up the patient carefully without missing timing of safe surgical treatment. In the borderline HCC patient, there is a risk to lose the chance of hepatectomy if the lenvatinib is not effective. In addition, there is a risk of poor healing of the wound in the following surgery [17].

Among HCC patients, about 6% patients were initially diagnosed with lung metastasis. Only 2.1% among these patients with lung metastasis received surgery [6]. Therefore, the reports were limited about the hepatectomy for primary HCC with synchronous lung metastasis. There are some reports that surgical treatment could achieve survival in selected patients [6, 18]. Surgical treatment in combination with lenvatinib therapy has a possibility to improve prognosis of HCC patients with lung metastasis.

According to pathological examination, although tumor showed broad necrosis, viable carcinoma still remained in the resected liver in this case. Because resected diaphragm and tumor of right portal vein did not have viable carcinoma, the patient showed good prognosis after the hepatectomy. Yamauchi et al. reported that tumor fibroblast growth receptor 4 (FGFR4) level before treatment was a predictor of response to lenvatinib [19]. If we performed a biopsy of the tumor at right portal vein transection operation, we might be able to evaluate the FGFR4 level of the tumor. FGFR4 immunohistochemistry in pretreatment tumor has a possibility to predict the response to lenvatinib therapy.

Recently, there have been reports that associating liver partition and portal vein ligation for staged hepatectomy (ALPPS) can also be performed for patients with HCC [20], which may also be applied for this case as well. However, we decided to transect the portal vein alone instead because we were concerned about the safety of ALPPS. It has been reported that future liver remnant volume hypertrophy takes an extended amount of time by portal vein dissection or embolism only. In actuality, this took 2 months in this case. However, in this case, this waiting time does not matter as the time was effectively used for lenvatinib administration.

## Conclusion

The treatment strategy from induction of lenvatinib to conversion hepatectomy including the portal vein transection was effective for a case of advanced hepatocellular carcinoma.

## Data Availability

Not applicable.

## Abbreviations

CT: Computed tomography
MRI: Magnetic resonance imaging
HCC: Hepatocellular carcinoma
PT-INR: Prothrombin time-international normalized ratio
PIVKA-II: Protein induced by the absence of vitamin K or antagonist- II
HBV: Hepatitis B virus
AFP: Alpha-fetoprotein
